# GMP-production of purified human B lymphocytes for the adoptive transfer in patients after allogeneic hematopoietic stem cell transplantation

**DOI:** 10.1186/s12967-017-1330-5

**Published:** 2017-11-07

**Authors:** Hannes Tittlbach, Andrea Schneider, Julian Strobel, Robert Zimmermann, Stefanie Maas, Bernd Gebhardt, Georg Rauser, Michael Mach, Andreas Mackensen, Thomas H. Winkler, Julia Winkler

**Affiliations:** 1 Department of Internal Medicine 5, Hematology/Oncology, University Hospital Erlangen, Erlangen, Germany; 2 Institute for Biology, Nikolaus-Fiebiger-Center for Molecular Medicine, Friedrich-Alexander-University Erlangen-Nuremberg, Erlangen, Germany; 3 Department of Transfusion Medicine and Hemostaseology, University Hospital Erlangen, Erlangen, Germany; 4 Center for Clinical Studies Erlangen, University Hospital Erlangen, Erlangen, Germany; 5 Miltenyi Biotec GmbH, Bergisch Gladbach, Germany; 6 Institute for Clinical and Molecular, Virology University Hospital Erlangen, Erlangen, Germany

**Keywords:** Adoptive immunotherapy, B cells, GMP-compliant manufacturing

## Abstract

**Background:**

We have recently shown that memory B cells from murine CMV immune donor animals adoptively transferred into immunodeficient mice were highly effective in protecting from a viral infection indicating a therapeutic potential of virus specific memory B cells. These preclinical data provided evidence that a cell-based strategy supporting the humoral immune response might be effective in a clinical setting of immunodeficiency after allogeneic hematopoietic stem cell transplantation. As adoptive transfer of B cells has not been used before in a clinical setting it was necessary to establish a technology for the generation of good manufacturing practice (GMP)-grade B cell products.

**Methods:**

Starting from the leukapheresis product of healthy blood donors, B cells were purified by two different separation strategies using GMP-grade microbeads and the CliniMACS system. A one-step protocol was used for positive enrichment of B lymphocytes with anti-CD19 microbeads. In a two-step enrichment protocol, first T lymphocytes were depleted by anti-CD3 microbeads and the remaining fraction was positively selected by anti-CD19 microbeads.

**Results:**

The purity and recovery after enrichment of B lymphocytes from the leukapheresis material in both separations strategies was not statistically different. However, contamination of the B-cell product with T cells was significantly lower after the two-step protocol (0.16%, range 0.01–0.43% after two-step separation and 0.55%, range 0.28–0.85% after one-step separation, p < 0.05). Therefore, a combined CD3 depletion and CD19 enrichment was used for the production of GMP-conform B-cell products from the leukapheresis material of 17 healthy stem cell donors. The absolute B-cell numbers obtained in the final product was 4.70 ± 3.64 × 10^8^ with a purity of 95.98 ± 3.31% B lymphocytes and a recovery of 18.9 ± 10.6%. Importantly, the contamination with CD3^+^ T cells was extremely low in the final B- cell products (0.10 ± 0.20%). Purified B cells exhibited normal antibody production after in vitro stimulation and showed excellent viability after cryopreservation.

**Conclusions:**

A GMP-grade B-cell product can be obtained with high purity and very low T-cell contamination using the two-step enrichment protocol based on CliniMACS® technology.

## Background

Patients after allogeneic hematopoietic stem cell transplantation (allo-HSCT) show a long-lasting immune deficiency involving both T and B lymphocytes [1]. Antibody responses to vaccination, as a measure of functional B-cell immunity, are insufficient in the first months after HSCT and recover usually within 1–2 years [2]. During the period of intensive immunosuppression the patients are highly susceptible to bacterial, fungal and, most importantly, viral infections. Among the viral pathogens that are major causes of morbidity and mortality human cytomegalovirus (HCMV) is one of the most challenging complication [3, 4].

As a persistent herpes virus, HCMV can ultimately be controlled only by a specific immune reconstitution. Thus, strategies to support HCMV-specific immune responses in the transplant recipient are actively pursued. Current immunotherapy approaches rely almost exclusively on the early restoration of recipient HCMV-specific immunity by adoptive transfer of cytotoxic CD8^+^ T cells from the donor (reviewed in [5]). This strategy has proven effective in the prevention of reactivation and treatment of HCMV infection that is unresponsive to antiviral therapy [6]. Sorting of virus-specific T-cell via multimers or via the IFN-γ selection is a well established procedure, meanwhile [7].

We have recently used the murine model of CMV (MCMV) as a preclinical model to investigate the potential of a novel cell-based strategy to support the humoral antiviral immune response. Memory B-lymphocytes from MCMV immune donor animals adoptively transferred into immunodeficient mice were able to protect from an ongoing viral infection indicating a therapeutic potential of virus-specific memory B cells [8]. These preclinical data provided evidence that a cell-based strategy supporting the humoral immune response might be effective in a clinical setting of post allo-HSCT immunodeficiency.

To improve immune reconstitution against CMV and other relevant pathogens, we developed a GMP-conform B-cell product for the adoptive transfer of memory B cells from the original stem cell donor. Here we describe the successful establishment of a two-step purification procedure for B lymphocytes that is performed under GMP conditions.

## Methods

### Donors and leukapheresis

Starting material for the B-cell enrichment were unstimulated mononuclear cell apheresis products (leukapheresis). Eleven B-cell separations were derived from eleven healthy donors enrolled in a study in order to establish the B-cell separation method. All donors gave written informed consent. The study protocol for these donors was approved by the local ethics committee (Nr. 3995) and the study was performed in agreement with the Declaration of Helsinki in its current version.

Seventeen leukapheresis products for the GMP-grade production of B cells used in the currently ongoing clinical phase I/II study (NCT02007811) were obtained from 17 donors who had previously donated the stem cell graft for an allo-HSCT. The study protocol was approved by the local ethics committee (Nr. 84_13Az) and the competent authority, the Paul-Ehrlich-Institut, Langen, Germany.

The leukapheresis procedure was limited to one day and was done using an apheresis system [either AS.TEC 204 (Astec, Fresenius Hemocare, Bad Homburg, Germany) or COBE Spectra (Gambro BCT BCT, Lakewood, Colorado)] processing 7–15 l, in accordance with the German guidelines of blood donation [9]. The volumes of the apheresis products ranged from 106 to 292 ml (median 194 ml). The absolute number of total nucleated cells in the apheresis products ranged from 7.3 × 10^9^ to 3.24 × 10^10^ (median 1.84 × 10^10^).

After collection, the apheresis products were stored overnight at room temperature (RT) on an orbital rotator (25 rpm) before processing.

### CD19 enrichment by one step immunomagnetic selection

The immunomagnetic selection of CD19^+^ B cells was based on the MACS® technology, (Miltenyi Biotec Bergisch-Gladbach, Germany) using the ClinicMACS® Plus device, one CliniMACS® tubing set LS (REF 162-01), the CliniMACS^®^ CD19 reagent (1 vial each) and three to four 1 l-bags of CliniMACS® PBS/EDTA buffer depending on the runtime at the CliniMACS® cell separator. Before separation 20% human serum albumin (HSA) (Baxter AG, Vienna, Austria) was added to the CliniMACS® PBS/EDTA buffer in a final concentration of 0.5% (w/v).

The following additional materials from Miltenyi Biotec GmbH were required: one 600 ml bag, one 150 ml bag, one sampling site coupler and two plasma transfer sets for the cell preparation procedure. Handling of the bags followed the instructions of the manufacturer as provided for the application of the CliniMACS® CD3 reagent and CD3/CD19 reagent combination.

The immunomagnetic selection of B cells started with the depletion of thrombocytes. For this purpose, the bag with the leukapheresis material was transferred to a 600 ml cell bag, which was then filled with CliniMACS® HSA buffer up to the weight of 600 g and centrifuged for 15 min at 300*g* without brake at RT. After removing the supernatant, the cell pellet was re-suspended and adjusted to a volume of 95 ml. Before labeling with anti-CD19 magnetic microbeads the thrombocyte-depleted fraction was incubated with 5 ml clinical grade intravenous immunoglobulin (ivIgG), (Kiovig®, Baxalta Deutschland GmbH, Unterschleißheim, Germany) for saturation of Fc receptors and processed on an orbital rotator (25 rpm) for 5 min at room temperature (RT). Directly after incubation, the CliniMACS® CD19 reagent was added to the product and incubated on the rotator (25 rpm) for another 30 min. To remove excessive reagent, the cell preparation bag was filled with separation buffer up to a weight of 600 g and centrifuged (300*g*, 15 min) with brake at RT. After centrifugation the supernatant was removed and the cell pellet was re-suspended and adjusted to a weight of 100 g. In accordance with the protocol from Miltenyi Biotec the CliniMACS® Tubing Set LS and the cell preparation bag was installed on the CliniMACS® device. Before starting the CliniMACS® device the following input parameters were entered: total number of cells (10^6^/ml), the volume of CD19-marked cell suspension (i.e. 100 g) and the relative proportion of CD19-positive cells using the measurement of the retained sample from the leukapheresis before thrombowash at the outset. Then enrichment program 1.1 was chosen. After the separation (lasting 30–45 min) the CD19-enriched target fraction was taken off the device in a 150 ml bag and a 1 ml samples for further analyses were taken.

### CD19 enrichment with two step immunomagnetic selection

The two step enrichment of CD19 B cells was based on the magnetic separation methodology from Miltenyi Biotec GmbH using the ClinicMACS® Plus device and two CliniMACS® LS tubing sets (REF 161-01), the CliniMACS® CD3 reagents (1 vial each) and the CliniMACS® CD19 reagent (1 vial each) and four to five bags 1 l CliniMACS® PBS/EDTA buffer, depending on the runtime on the CliniMACS® cell separator. The following additional materials from Miltenyi Biotec GmbH were required: six 600 ml bags, one 150 ml bag, three sampling site couplers and 4 plasma transfer sets for the two step cell preparation procedure.

The CliniMACS® PBS/EDTA buffer was supplemented with human serum albumin (Baxter AG, Vienna, Austria) to a final concentration of 0.5% (w/v) and the depletion of thrombocytes from leukapheresis product was performed as described above.

After removal of the supernatant and re-suspension of the cell pellet the thrombocyte-depleted cell fraction was adjusted with buffer to the volume of 90 ml. Before labeling with anti-CD3 microbeads clinical grade ivIgG was added to the cell suspension as described above. One vial of 7.5 ml of CliniMACS® CD3 reagent was added to the product which was then incubated on the rotator (25 rpm) for 30 min.

One vial of anti-CD3 reagent is sufficient for the depletion of up to 15 × 10^9^ CD3 positive cells out of a total cell number not exceeding 40 × 10^9^ white blood cells. For labeling preparations exceeding these thresholds, two vials of CD3 reagent were required.

After incubation, the cell preparation bag was filled with separation buffer to 600 g and then centrifuged (300*g*, 15 min) with brake at RT. After centrifugation the supernatant was removed and the cell pellet was re-suspended and adjusted to a volume of 150 g. Before starting the CliniMACS® device the following parameters were entered into the software: total cells (10^6^/ml), the volume of CD3-marked cell suspension (i.e. 150 g) and the relative proportion of CD3-positive cells using the measurement of the retained sample. Depletion program 2.1 was chosen. Then the CliniMACS® Tubing Set LS and the cell preparation bag were installed on the CliniMACS® device in accordance with the protocol from Miltenyi Biotec. After the procedure (lasting 1.5–3 h) the tubing set and all of the bags were disconnected but only the bag with the CD3-depleted fraction, the target-fraction was used for the following CD19 enrichment. Before starting the CD19 enrichment a 1 ml sample was taken for flow cytometry.

For the CD19 enrichment the bag containing the CD3-depleted cell suspension was filled with the buffer up to the weight of 600 g and centrifuged (300*g*, 15 min) with brake at RT. After removing the supernatant the cell pellet was resuspended and adjusted to a volume of 95 ml.

The subsequent labelling of the CD19-positive cells and separation with the CliniMACS® device was performed exactly as described above. The parameters for the separation were obtained from a sample from the target fraction after CD3 depletion.

### Clinical scale B cell selection

The clinical grade B cell selection was a two-step separation performed in a GMP-compliant laboratory (Department of Transfusion Medicine and Hemostaseology, University Hospital Erlangen, Erlangen, Germany) including first a CD3 depletion followed by CD19 enrichment using a GMP conform closed system from Miltenyi Biotec GmbH (Bergisch-Gladbach, Germany). For flow cytometry analysis and microbiological tests we took a 1 ml sample of each cell fraction. The following fractions were obtained: (1) leukapheresis before thrombowash; (2) after thrombowash; (3) after binding of CD3 beads; (4) target fraction after CD3 depletion; (5) non-target fraction after CD3 depletion; (6) after binding of CD19 beads; (7) target fraction after CD19 enrichment; (8) non-target fraction after CD19-enrichment.

### Flow cytometry

Leucocyte concentration in the starting population, CD3-depleted target fraction and CD19-enriched fraction were determined using the Sysmex XT1800 automatic hemocytometer (Sysmex Deutschland GmbH, Norderstedt, Germany). Flow cytometry analysis was performed on a FACS Calibur (Becton–Dickinson, Heidelberg, Germany). Cells were stained for 30 min at RT with a mixture of the following antibodies in a final concentration: 1:20 CD20-FITC (LT20), 1:100 CD14-PE (TÜK4), 1:40 CD15-PE (VIMC6), 1:100 CD3-APC (WW264-56) from Miltenyi Biotec GmbH (Bergisch-Gladbach, Germany) and 1:40 CD45-PerCP (2D1) from BD Biosciences GmbH (Heidelberg, Germany).

Because of it high cell number in the fraction 1–4 these fractions were diluted 1:10 before staining. For flow cytometry, 50 µl samples from each cell fractions were used, except for the CD19-enriched target fraction for which 200 µl was used to acquire a sufficient number of cells for the reliable quantitation of residual T cells.

For every measurement, BD TruCOUNT™ tubes (BD Biosciences, Heidelberg, Germany) for determination of absolute cell numbers were used. To assess cell viability, 7-AAD (BD Biosciences, Heidelberg, Germany) was added to the FACS suspension puffer. After staining, erythrocytes were lysed with a 1:10 prediluted lysis solution (BD Pharm Lyse™, Heidelberg, Germany) and the sample was filled up to 1 ml with the lysing solution. The probe was ready for measurement after incubation of 5 min at RT.

### Cryopreservation and thawing

After separation, the target fraction was divided into portions with different cell doses according to the specifications of the clinical trial. Each portion to be frozen was transferred into a cryobag inside a laminar flow cabinet situated in a clean room, depending on the product volume (Cryocyte freezing container R4R9951, R4R9953 or R4R9955, respectively, Baxter Healthcare Corp., Deerfield, IL). Subsequently, a solution of 20% HSA (Baxter AG, Vienna, Austria) was added to the B-cell product in at a ratio of 1:1. The cryobags with the B-cell product were cooled to 4 °C prior to the addition of the cryoprotective agents. As cryoprotective agent a solution of 70% dimethyl sulfoxide in sodium chloride (DMSO-NaCl) was subsequently added in a ratio of 1:10 (vol/vol) to each B-cell product to achieve a final DMSO concentration of 7%. In each case, a 500 µl aliquot of the final B-cell suspension was transferred into a sterile polypropylene cryovial (Cryo tube vials, 1.8 ml, Nunc A/S, Roskilde, Denmark). Cryobags and cryovials were simultaneously frozen at a controlled rate to a final temperature of − 100 °C within 62.5 min using a liquid nitrogen cryopreservation chamber (Biofreeze BV50, Consartic, Schoellkrippen, Germany) and afterwards stored below − 150 °C in the vapor phase of liquid nitrogen until analysis [10].

The cryovials were thawed at 37 °C for about 30 s until ice crystals completely disappeared. Immediately afterwards, thawing medium in a 1:3 v/v dilution was added, containing nine parts dextran-40 and one part 5% HSA [10]. Samples for viability analysis were then taken directly without centrifugation and additional washing of the cells.

### In vitro B-cell activation assay

In vitro stimulation of B-cell preparations was performed under limiting dilution conditions essentially as described in Poetzsch et al. [11]. In brief, cells were sorted at different cell numbers/well directly into 96 well microplates containing a confluent layer of irradiated feeder cells (human foreskin fibroblasts), using a MoFlo cell sorter (Cytomation, Germany). Sorted cells were grown in complete RPMI-1640 medium supplemented with 2 mM glutamine, 100 IU/ml penicillin, 100 mg/ml streptomycin, 50 mM 2-mercaptoethanol and 10% FCS (heat-inactivated) (PAN-Biotech, Germany) in the presence of Epstein-Barr-Virus (EBV) and CpG ODN 2006. After 3 weeks, the culture supernatants were screened for IgG secreted in the supernatant by ELISA.

### Statistical analysis

Statistical analysis included descriptive statistics and correlation coefficients using the Wilcoxon-Mann–Whitney-Test as appropriate. A p-value of less than 0.05 was considered significant. Statistical analysis was performed using statistical software (GraphPad Prism, GraphPad Software, Inc., San Diego, California).

## Results

### Comparison of one-step and two-step separations

**Fig. 1:**
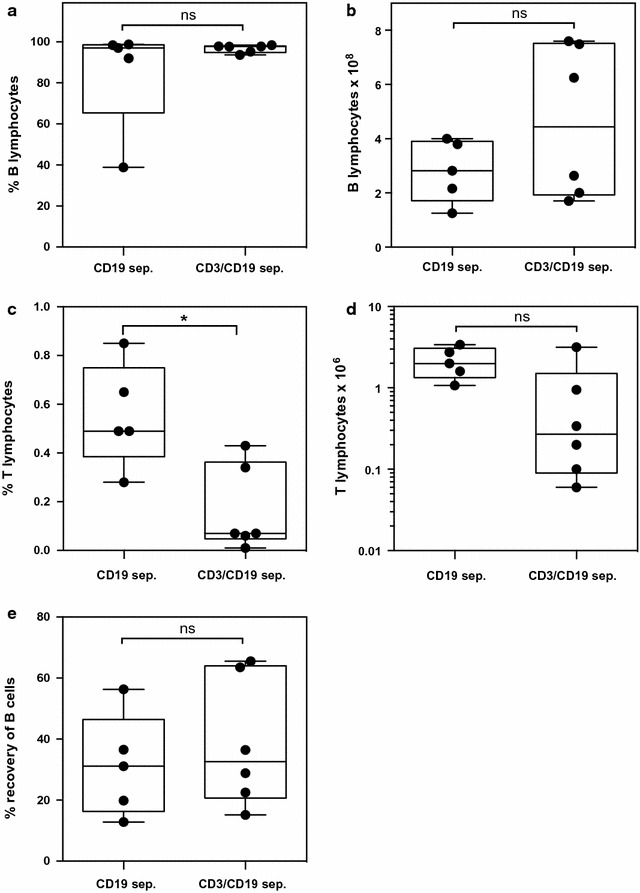
Comparison of one-step and two-step separation strategy. **a** Purity of CD19^+^ B cells in %; **b** absolute yield of B cells; **c** contamination of B-cell product with CD3^+^ T-lymphocytes in %; **d** absolute numbers of T-lymphocytes in the B-cell product; **e** recovery of B-lymphocytes in % of the starting leukapheresis material. Box plots figures for one-step (CD19^+^ selection; n = 5) and two-step (CD3^−^ selection followed by CD19^+^ selection; n = 6) separations are shown; *p < 0.05 (Mann–Whitney), ns not significant

To first optimize the purity and yield of B cells we performed five one-step separations and six two-step separations with leukapheresis material on the CliniMACS under non-GMP conditions. The one-step separation comprised a single positive selection with CD19-beads and the two-step separation started with a depletion of CD3^+^ T-cells and subsequent positive separation of CD19^+^ B cells also by magnetic beads on the CliniMACS device. Similar purity was obtained with both separations strategies with one exception for the one step purification with a median of 97.0% (range 38.9–98.7%) after the one-step enrichment and 97.7% (range 93.6–98.4%) after the two-step protocol (Fig. 1a). Absolute numbers of B cells were also similar (Fig. 1b). Whereas the purity of B cells was not significantly different, the percentage of contaminating CD3^+^ T cells after separations was significantly lower with the two-step protocol (median 0.07%, range 0.01–0.43% after two-step separations and median 0.49%, range 0.28–0.85 after one-step separations, p = 0.013; Fig. 1c). In line with this, the absolute numbers of T cells in the preparations were lower after two-step separations (Fig. 1d). The recovery of B lymphocytes was comparable with both separation strategies (median 31.1% after one-step separations and 32.6% after two-step separations; Fig. 1e). As contamination with T cells is of critical importance to avoid GVHD reactions, the two-step separations for the GMP-production of purified human B lymphocytes were used.

### Functionality of B cell in vitro

To evaluate the potential functionality of the purified B cells in vitro, we stimulated the isolated B cells with CpG under limiting dilution conditions as described by Poetzsch et al. [11]. As shown in Table 1 frequencies of IgG-producing B cells after stimulation ranged from 1:8 to 1:368 for single-step isolations and from 1:5 to 1:135 for two-step isolations. The variations presumably result from different percentages of IgG-positive memory B cells within the donor B cells. For one B cell product derived from a HCMV-positive donor we were able to test HCMV glycoprotein B (gB) and tetanus toxoid (TT) specific antibodies secreted by IgG producing B cells in our limiting dilution assay. The frequencies for IgG anti-HCMV-gB and anti-TT B cells were 1/1.450 and 1/1.600, respectively. Importantly, the purified B cells are fully functional for antibody production after stimulation in vitro.

**Table 1:** Frequencies of IgG-secreting B cells after stimulation of CD19 oder CD3/CD19 separated B cells with CpG in the limiting dilution assay

Number	Separation	Frequency of IgG-secreting B cells
1	CD19	1:368
2	CD19	1:8
3	CD3/CD19	1:7
4	CD3/CD19	1:135
5	CD3/CD19	1:5
6	CD3/CD19	1:35
7	CD3/CD19	1:68

Frequencies of IgG-secreting B cells after stimulation of CD19 oder CD3/CD19 separated B cells with CpG in the limiting dilution assay

### B cell enrichment under GMP conditions

For the production of clinical grade B cells, we manufactured B cell products under GMP conditions from 17 unstimulated leukapheresis products derived from the original stem cell donor. Mean absolute number of CD45-positive leukocytes before separation was 184.39 ± 73.08 × 10^8^ and the mean absolute number of B cells was 24.40 ± 14.80 × 10^8^ (Table 2). Purities of CD20^+^ B cells (median of 97.1%, range 85–99%) with only very low frequencies of contaminating T cells (median of 0.03%, range 0.01–0.82%; Table 2) were achieved. The yield of B-lymphocytes obtainable from a single leukapheresis product after the two-step separation was 4.70 ± 3.64 × 10^8^ B lymphocytes. Based on a body weight of 70 kg of the recipient, we achieved mean B cell numbers of 6.71 × 10^6^/kg b.w. (range 0.57–18.97 × 10^6^/kg b.w.).

**Table 2:** Summary of GMP separations

Number	Fractions	CD45+ cells (× 10^8^)	CD19+ cells (%)	CD19+ cells (× 10^8^)	CD3+ cells (%)	CD3+ cells (× 10^8^)	Recovery of B cells (%)^a^
1	Leukapheresis before thrombowash	184.5 ± 73.08^b^	13.62 ± 5.27	24.40 ± 14.80	49.01 ± 6.41	87.76 ± 38.36	100
2	After thrombowash	161.84 ± 66.92	13.2 ± 4.93	21.19 ± 13.25	49.02 ± 12.85	78.28 ± 35.79	89.6 ± 16.8
3	After binding of CD3 beads	114.18 ± 65.94	17.2 ± 6.19	17.37 ± 9.33	32.35 ± 15.78	37.18 ± 37.27	80.5 ± 37.3
4	Target-fraction after CD3 depletion	58.25 ± 37.62	24.63 ± 10.47	13.18 ± 10.0	0.16 ± 0.27	0.23 ± 0.70	50.4 ± 17.3
5	Non-target fraction after CD3 depletion	43.81 ± 29.64	10.06 ± 5.99	3.15 ± 1.94	57.14 ± 22.08	27.45 ± 25.39	16.4 ± 10.6
6	After binding of CD19 beads	48.65 ± 32.57	17.77 ± 6.98	6.26 ± 4.58	0.14 ± 0.28	0.07 ± 0.13	27.1 ± 13.4
7	Target-fraction after CD19 enrichment	5.04 ± 3.77	95.98 ± 3.31	4.70 ± 3.64	0.10 ± 0.20	0.005 ± 0.006	18.9 ± 10.6
8	Non-target fraction after CD19 enrichment	33.42 ± 14.49	0.34 ± 0.45	0.10 ± 0.15	0.16 ± 0.33	0.07 ± 0.13	0.39 ± 0.68

^a^Recovery of B cells relative to fraction 1

^b^Mean ± SD of 17 separations

**Fig. 2:**
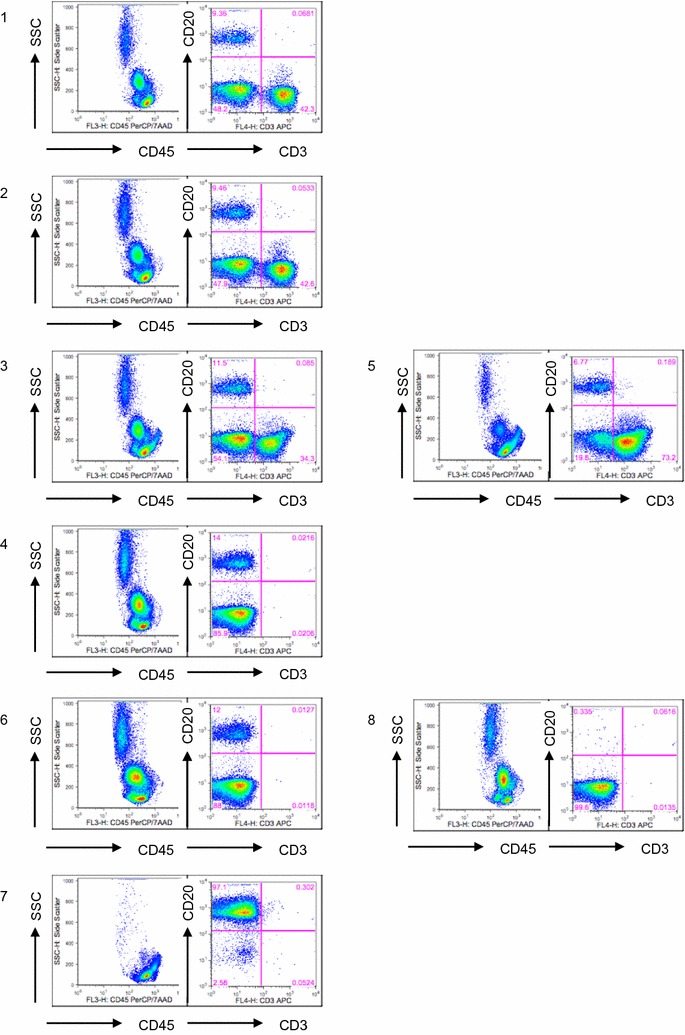
Immunophenotype of individual fractions from the two-step separation protocol. Density plots of combined stainings with CD45 PerCP, CD20-FITC and CD3-APC antibodies. **1** leukapheresis before thrombowash; **2** after thrombowash; **3** after binding of CD3 beads; **4** target fraction after CD3 depletion; **5** non-target fraction after CD3 depletion; **6** after binding of CD19 beads; **7** target fraction after CD19 enrichment; **8** non-target fraction after CD19 enrichment. Flow cytometry density plots of one representative separation performed under GMP conditions are shown

**Fig. 3:**
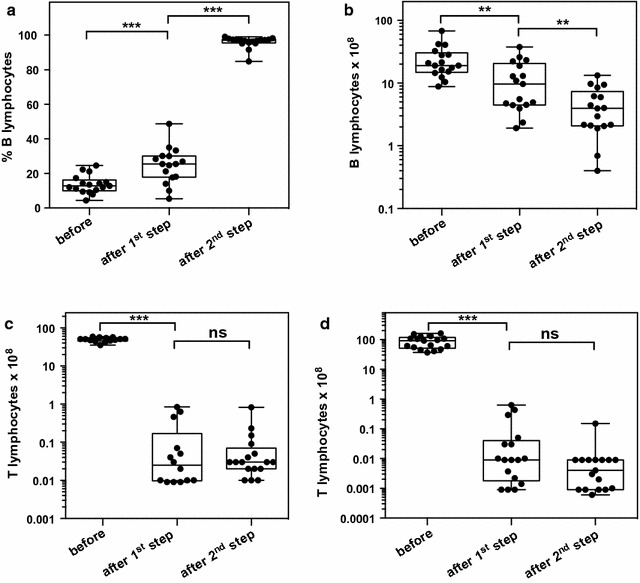
B-lymphocyte separations performed under GMP conditions. Summary of characteristics of 17 B-cell separations performed from leukapheresis material from the original stem cell donor under GMP conditions. **a** Purity of CD19^+^ B cells in %; **b** absolute number of B-lymphocytes **c** contamination by CD3^+^ T-lymphocytes in % **d** absolute number of T lymphocytes. **p < 0.01, ***p < 0.001 (Mann–Whitney)

In Fig. 2 a representative flow cytometric analyses of the separation steps are shown and the data are summarized in Table 1. As can be seen from the analysis of fractions 3 and 4, T cell depletion was generally very efficient with a remaining contamination in the first target fraction (fraction 4 in Table 2) of 0.16 ± 0.27% (mean ± SD). The non-target T cell fraction, however, contained significant percentages of B cells in all isolations (mean 10.06 ± 5.99%). The frequency of B cells in the non-target T-cell fraction comprised 16.4% of the input B cells in the mean and will be discussed below. The target B cell fraction after CD19-positive selection contained up to 99.1% CD20^+^ B cells (mean 95.98 ± 3.31, Table 2) and contaminations with CD3^+^ T cells were further reduced (0.10 ± 0.20%). In the non-target flow through material that contained mainly myeloid cells B cells were detected only at very low frequencies (0.34 ± 0.45%, Fig. 2 and Table 2). The overall recovery of B cells was somewhat lower as compared to the test runs with a mean percentage of 18.9 ± 10.6% of the input leukapheresis material for all 17 GMP productions (range 2.3–49.5%, Table 2 and Fig. 3).

### Quality control of B cell product

For each of the clinical grade B cell preparations aliquots were taken for microbiological tests and for thawing the cells and assessing cell viability. Despite extended manipulations of the cells, sterility of the B cell products was proven in all cases. The viability of the B cells after cryopreservation was high, ranging from 92.2 to 98.4% (mean 95.9 ± 2.0%) after thawing.

## Discussion

As the reconstitution of a functioning immune system after allogeneic HSCT takes months to years [1], the adoptive transfer of memory B cells from the donor might be a new strategy to overcome the immunodeficiency observed in the patients for extended periods of time [8]. In this study, we show for the first time the feasibility of the production of a clinical grade B cell product from leukapheresis material from stem cell donors for such an adoptive transfer into patients. These results provide the prerequisite for the clinical evaluation of the new concept of transferring the complete B cell memory of the donor to the HSCT patient.

Our data provided here show that it is feasible to generate a cellular product containing on average 5 × 10^8^ highly pure B cells using a two-step separation technology on a CliniMACS device. This included recruitment of the original stem cell donor for leukapheresis, transport and overnight storage into the GMP laboratory and purification. The most important quality measure of a B-lymphocyte product for adoptive immunotherapy after HSCT is the contamination with T-lymphocytes as potential inducers of graft versus host disease (GvHD). We therefore intended a maximum acceptable contamination with CD3^+^ T cells of 4 × 10^4^ T-cells/kg bodyweight of the recipient, which is the critical threshold number of T-lymphocytes in haploidentical HSCT [12]. We reliably achieved this very low level of T cell contamination with a two-step separation protocol.

The detailed monitoring of the process of cell purification revealed a significant number of B cells within the non-target fraction after CD3 depletion. We consider two possibilities for this unexpected loss of B cells in the non-target fraction. First, Fc-receptor-mediated binding of B cells [13] to the anti-CD3 antibody coated microbeads might lead to the loss of B cells. We tried to overcome this binding by adding clinical grade human IgG preparations to saturate Fc receptors. Increasing of the IgG concentration could potentially further reduce binding. Alternatively, future studies could make use of the recently introduced CliniMACS TCRαβ Biotin Reagent [14] that might have less binding to B cells. As a second possibility, T cell–B cell doublets or even multimers after insufficient resuspension of the cells in the preparation bags after centrifugation might be responsible for the loss of B cells in the non-target fraction. Automated cell processing including centrifugations in the CliniMACS Prodigy system might be able to reduce the formation of doublets.

As the adoptive transfer of memory B-lymphocytes from the HSCT donor into patients after allogeneic stem cell transplantation has not been tested before in a clinical setting, it is difficult to predict whether the B cell numbers that we were able to obtain by the current protocol described in this paper will be sufficient to mediate significant protection against viral infections including HCMV, Adenovirus, BK virus or HHV6. With the following assumptions and extrapolating from animal experiments, however, we can calculate a minimum number of memory B cells required for a significant antibody response. The frequency of virus-specific memory B cells among total B cells in adults was measured to be approximately 1:5.000 with some variations in donors and among specificities [11, 15, 16]. Our cellular product containing 5 × 10^8^ B cells would include about 1 × 10^5^ virus specific memory B cells against the viruses the donors was immunized against. We have shown previously in the mouse, that 25 sorted CMV-specific B cells are sufficient to mount a robust antibody response after antigenic challenge [17]. Extrapolating the body weight of a mouse (20 g) to an average of 70 kg of the patient it can be concluded that sufficient memory B cells would be contained in a B cell product to achieve similar responses in patients.

The separation strategy that we used here selected total B cells primarily because the only GMP-grade magnetic beads for B cells were CD19 beads. The development of CD27 magnetic beads would allow the purification of CD27^+^ memory B cells. As the depletion of CD3^+^ cells in the first step of magnetic bead selection was usually extremely good, a second positive selection with CD27 beads could be suitable to purify memory B cells, despite the expression of CD27 on a substantial number of T cells. Alternatively, future developments could allow FACS cell sorting of CD19^+^ CD27^+^ memory B cells on clinical grade FACS cell sorters.

Currently, it is not possible to expand in vitro human memory B cells for the application in the clinics. Such expansion protocols would potentially overcome the high cellular input necessary for the adoptive therapy with memory B cells. Culture systems developed in the mouse are able to expand B cells based on signals provided by CD40, IL-4, BAFF and IL-21 [18]. As these cells can confer long term memory in vivo [18], similar protocols should be established for human B cells under GMP-qualified conditions for clinical use.

Also in haploidentical stem cell transplantation reconstitution of B cells is severely delayed. This has been described for protocols using T cell depletion of the graft as well as for post-transplantation cyclophosphamide protocols for the first 6 months posttransplantation [19]. Therefore, the adoptive transfer of allogeneic B cell preparations from the donor could also be considered for reducing the risk of infections in these patients.

With the protocol described in this paper a manufacturing license according to the German Medicinal Products Act (AMG) was obtained. We are currently investigating the safety, tolerability and feasibility of an adoptive transfer of allogeneic B cell preparation after allo-HSCT in a phase I/IIa clinical trial (ClinicalTrial.gov NCT02007811). In this first-in-man clinical trial in 4 groups with escalating doses the primary outcome measures are EBV reactivation, post-transplant lymphoproliferative disorder associated with EBV reactivation and development of acute or chronic graft-versus-host-disease. Results from this ongoing trial will provide important data for the potential future application of adoptive transfer of memory B cells potentially being beneficial for a variety of infectious pathogens in patients after allo-HSCT.

## Conclusions

A method for the GMP-compliant production of B cells from leukapheresis material was established. With a two-step separation on a CliniMACS device high purity of B cells and minimal T cell contamination was achieved. The absolute numbers of B cells obtainable by this method might be sufficient for adoptive cell therapy to support the immunodeficiency of patients after allo-HSCT.

## Abbreviations

allo-HSCT: allogeneic hematopoietic stem cell transplantation
EBV: Epstein-Barr-Virus
GMP: good manufacturing practice
HCMV: human cytomegalovirus
MCMV: murine cytomegalovirus
